# Leucoplakia—Abstract

**Published:** 1898-10

**Authors:** J. S. Marshall


### THE DENTAL REGISTER.

VOL. LII.] OCTOBER, 1898. [No. 10.

### COMMUNICATIONS.

### Leucoplakia—Abstract.

BY J. S. MARSHALL, M. D.

Read before the Stomatological Section of the American Medical Association, at Denver, June, 1898.

Leucoplakia is a term derived from the Greek *Lukia*, white, and *Plakia*, surface—literally white surface, or whitening of the surface.

DELINEATION: Leucoplakia is a chronic, superficial inflammation of the mucous membrane of the tongue, the palate, the cheeks and the gums, and is characterized bv the presence of pearly-white or bluish-white plaques or patches; in some cases small, others covering the entire dorsum of the tongue, the cheeks from the angle of the mouth back to the fauces, the palate or the entire buccal surface of the gums.

NOMENCLATURE: The disease is variously known as psoriasis linguae, zona (herpes zostal), smokers' patch, leucoma, leucoplakia, ichthyosis, leucokeratosis, leucoplasis, leucoplaques, plaques opaline and superficial glossitis.

VARIETIES: There are two forms of leucoplaques found in the human mouth: the milky, opaline patches (plaques opaline), represented bv the mucous patches of condylomata of secondary syphilis, and the non-syphilitic, smooth, white or pearly-white patches for which Schwemmer was the first to propose the leucoplakia, and Hutchinson leucoma.

The plaques opaline or the mucous patches of secondary syphilis, are grayish-white and curdy in appearance, resembling the superficial corrosions caused by the application of the nitrate of silver to the mucous membrane; while the plaque of leucoplakia are usually thin, shiny, bluish-white, white or pearly in color, sometimes having a yellowish tinge, but this, according to Butlin, is almost always due to the stain of tobacco or some other extraneous substance.

These two varieties of leucoplaques may be further differentiated by the slight elevation of the syphilitic mucous patches, the secretion of a thin, watery fluid, which is the potent source of contagion, and their tendency to become painful and to ulceration; while in leucoplakia the patches are not elevated above the surrounding tissue except in the warty form (ichthyosis); they are not painful except in the advanced stage of the disease, no secretion is present, and ulceration is not developed until the disease has taken on a malignant form.

To the latter variety of leucoplaques, the true leucoma or leucoplakia, the essayist desires at this time to call your especial attention for the following reasons:

First. It is an exceedingly dangerous affection, often being the forerunner of carcinoma.

Second. It is a disease which, from its innocent appearance and the painless character of its early stages, is seldom recognized until the disease has progressed to a stage which renders a favorable prognosis exceedingly doubtful.

Third. That the disease seems, from personal observation, to be on the increase.

Fourth. The dentist, from the very nature of his specialty, is in a position to see and recognize the disease in its earliest stages, and to warn the patient of his condition before it has progressed so far as to prove a menace to his life.

The disease in its early stages is much more likely to come under the notice of the observing dentist or stomatologist than it is of a surgeon or the laryngologist. As a rule, the patient does not consult a surgeon until the disease becomes troublesome. The dentist, therefore, should be so familiar with the characteristic features of the disease that he could recognize it at a glance, while it would be his duty to impress upon the patient the urgent necessity of consulting an oral specialist with the view of instituting measures calculated to arrest its further development, or for its complete extirpation.

Etiology: The etiology of leucoplakia is by no means a settled question in oral pathology.

The early writers looked upon the disease as a local manifestation of psoriasis, others that it was due to certain forms of skin disease, like zona (herpes zosta or hives), and lichen planus; many have looked upon the disease as a circumscribed chronic inflammation of the oral mucous membrane due to syphilis, and still others that it is a distinct affection produced by the local irritation induced by smoking or chewing tobacco.

Various inflammations and erupive diseases of the skin, but which often extends to the mucous mmbrane, especially of the mouth and nose, while upon the other hand it may originate in some wound or inflammation of the mucous membrane and later extend to and involve the skin.

Zona is another example in the same line. Although zona is an eruptive disease of the skin it often attacks the mucous membrane of the lips and of the genital organs at the junction of the skin with the mucous membrane.

Lichen planus, another skin affection, sometimes produces buccal lesions. These lesions have been described by Wilson, Hutchinson, Kaposi and Crocker as whitish, thickened and uniformly elevated plaques upon the mucous membrane surface, sometimes grayish-white or resembling in color the places which have been cauterized with nitrate of silver.

It is not strange, therefore, that the early writers should look upon loucoplakia as a manifestation of some of these forms of skin diseases, and particularly of psoriasis, which it somewhat closely immulates in its earlier stages.

Most modern writers look upon leucoplakia as an entirely distinct affection, having no association with psoriasis in any of its forms. Hyde says: "Psoriasis is not known to affect the mucous surfaces. The lesions of so-called psoriasis linguae are those of leucoplakia buccalis, of smokers' patches, of syphilitic disease of the mouth, or of flat epithelioma."

Nicholson, however, still holds to the old theory that leucoplakia is a local manifestation of skin affection, and maintains that the disease is zona (herpes zosta), located in the mucous membrane. The peculiar burning sensation that accompanies the white patches located upon the lingual mucous membrane he considers as almost a pathognomonic sign, and calls attention to the fact that one or two herpetic vesticles may appear on the lower surface of the tongue during the course of the disease. Park is of the opinion that leucoplakia is often due to syphilis, and says: "These late and recurring lesions (syphilitic mucous patches lose their moist character, become quite smooth, shiny, of a bluish-white color, and may mark the beginning of the condition known as leucokeratosis."

Predisposing Causes: Butlin agrees with DeBove in the statement that there is, in most patients, some condition which predisposes to the disease. He says: "I suspect that the mucous membrane of the tongue in leucomatous subjects is from the first less thick and stable, and more easily irritated than in the majority of persons. As some persons are known to have irritable and delicate skins, easily inflamed and prone to eruptions, and as some of these persons develop affections of the skin which are very chronic and difficult to heal, so I believe other persons have a tongue whose mucous membrane is abnormally delicate, prone to chronic inflammation, and difficult to cure when the disease has been excited. It has been suggested that chronic dyspepsia and the rheumatic and gouty diathesis might be a predisposing cause of the disease, but the evidence upon this point does not seem to be sufficiently strong to give any real weight to its consideration."

Age and sex are both very important factors in the causation of the disease. Leucoplakia has been rarely seen in persons under twenty years of age, even in boys addicted to smoking; while upon the other hand, it is rarely seen to commence in persons over sixty years of age. Women seem to be almost entirely exempt from the disease. Of the eight cases seen by the essayist, all were in men, and occurred between the ages of fifty and seventy-four. Du Castel has reported a case in which the disease had existed since the age of twelve years in a man who had never used tobacco.

Exciting Causes: Among the most common exciting causes of leucoplakia may be mentioned the irritation produced by the habitual use of tobacco, particularly smoking, the latter recurring lesions of the mucous membrane due to the secondary manifestations of syphilis; the mucous plaques, acting locally upon the tongue or the buccal mucous membrane; the frequent use of undiluted spiritous liquors; the drinking of very hot fluids or eating of very hot or highly spiced foods, the mechanical irritation of teeth roughened by the process of caries, fractures or the accumulation of salivary calculus; the irritation from dental plates which are rough, ill-fitting or made of material which is irritating to the delicate mucous membrane of the mouth.

Wallenberg is of the opinion that the use of tobacco is the most frequent source of leucoplakia, and believes the disease is produced by the irritation of the volatile and empyreumatic oils, liberated in smoking it. The essayist has no hesitation in expressing it as his opinion that the use of the pipe is much more dangerous to a sensitive mouth on this account than the smoking of cigars or cigarettes, as the pipe, from long use, is generally saturated with these oils, which often come into direct contact with the mucous membrane of the tongue, causing smarting and burning sensations with more or less irritation. In the habitual smoker the irritation becomes chronic, producing a thickening of the epidermal layer and infiltration of the papillary layer with round cells. Erb collected and analyzed 240 cases of leucoplakia, and states as his belief that the lesions are, as a rule, due to "epithelial thickening, resulting from syphilitic mucous patches." Of this number two only were in women, and these were both syphilitic.

In about 80 percent of the cases there was a clear history of syphilis, while in many of the others a very strong suspicion existed of such an affection. Anti-syphilitic treatment in four or five cases either cured or greatly improved the conditions, even when they had existed for a long time. Out of 148 cases who were interrogated as to their use of tobacco, forty-seven smoked little or not at all; 101 smoked moderately, and two excessively. Syphilis alone occurred in thirty-six of these cases, smoking alone in thirty-seven; syphilis and smoking occurred in sixty-four, and neither in eleven cases. The following conclusions were reached: 1st. Syphilis or smoking alone may be the cause of this affection in about the same proportion of cases. 2d. In a majority of cases it may be due to both. 3d. It rarely appears without reference to one or the other of these causes. 4th. Other forms of irritation seem to play only a minor part. He believes that a certain predisposition must be assured in view of the great number of syphilitic smokers who never developed the disease.—(Sarjous' Annual.)

SYMPTOMS AND DIAGNOSIS: Leucoplakia may be recognized by the presence of circumscribed or diffuse smooth white, bluish-white or pearly-white radiating patches appearing in varying numbers upon the mucous membrane of the cheeks, lips, gums, palate or the tongue. These patches often coalesce to form larger ones. In their earliest stage they are not elevated above the surrounding membrane, are smooth and glistening in appearance, and range in size from tiny, irregularly outlined spots to large plaques of a silver half dollar, or even larger. At first they are not sensitive, and on this account may exist for a long time without the knowledge of the patient. Many cases never progress beyond this stage. Others may slowly increase in size, thickness and intensity of color, the plaque being slightly raised, the surface hard, cornified and roughened. Accompanying this stage, especially when the disease is located upon the dorsum of the tongue, the patient will complain of persistent dryness of the parts and inability to use the tongue with comfort, except by frequent moistening of the mouth.

Later fissures appear in the tongue, and there is developed a smarting, burning sensation, as though the parts had been scalded. Alcoholic liquors, fermented beverages, acid fruits, highly seasoned or very hot foods or drinks, and chewing and smoking tobacco increase these sensations, and sometimes render the partaking of food a very great discomfort. Associated with this condition there is a tendency of some portion of the plaque to peel off or slough out from time to time, leaving a reddened or raw surface, which is exceedingly sensitive, sometimes quite painful. Ulceration may follow, and degenerative changes develop ending in the formation of a carcinoma.

When the disease is in the tongue, warty growths appear in the leucomatous patches, which show a marked tendency under the stimulation of an irritant to take on a rapid form of carcinomatous degeneration.

Another fact should also be borne in mind in diagnosing this affection, viz: the progress of the disease is, in many instances, very slow, and may have the appearance of having reached the limits of its development, while occasionally the disease may disappear with advancing age. On the other hand, the disease, which has seemed for many years to remain in about the same condition, may suddenly assume a most rapid and malignant type of degeneration.

Shield reported a case of leucoplakia linguae in a man seventy-three years old, for whom, two years before, one-half of the tongue had been removed for undoubted carcinoma, who gave a previous history of the disease, "bad tongue," for more than twenty years.

Differential Diagnosis: The affections which may be confounded with leucoplakia buccalis are the muco-plaques of syphilis and epithelioma. In the earlier stages of the disease such a mistake could hardly be made, but in the later period of the affection it might quite easily be confounded with syphilis or epithelioma. A three or four weeks' course of treatment with the iodide of mercury or potassium would clear up the diagnosis of the former, while in the latter it would be necessary to resort to the aid of the microscope for a positive diagnosis, even.

Pathology: In examining the histologic structure of the leucomatous patches, whether thick or thin, a marked change will be noticed in the character of the papillary layer of the tongue, the mucous of the lips and the cheeks, and in the cells of the epidermis.

The papillae of the tongue are often very much atrophied, and occasionally have almost entirely disappeared; while the epidermal layer has taken on a horny character more like that of the skin. This is true also of the epidermal layer in leucoplakia of the mucous membrane of the lips and of the cheeks. It is also noticed that the epithelial processes, both of the tongue and of those portions of the oral mucous membrane affected by leucoplakia are much shorter than is natural, and that the corium is infiltrated with leucocytes.

In the advanced stages of the disease true cell-nests are discovered, which establishes the fact of carcinomatous degeneration. How these cell-nests are formed in carcinoma is still a disputed question, but it would seem more than probable that in carcinoma of the tongue and oral mucous membrane following leucoplakia the *cell-nests were developed from traumatic inclusions of epethelial cells following the repeated ulceration and healing of the leucomatous patches*, though there was present the clinical evidence of enlarged lymphatic glands.

Pathology—Prognosis: The interest in the prognosis of leucoplakia centers around the tendency or the predisposition of the disease to be followed by malignant degenerative changes, ending in the formation of carcinoma. That such a predisposition exists there is not a shadow of a doubt.

In the American Text-Book of Surgery we find this statement: "Many cases of cancer of the tongue are preceded by leucoma, the so-called psoriasis of the tongue." Garretson was of the opinion that the disease occupied the border line between the non-malignant and the malignant growths. Le Dentu says: "It is not at all unusual for leucoplakia to become epitheliomatous." He does not, however, consider this to be a general predisposition of the disease, but that it is sometimes induced by a tendency of leucoplakia to degeneration. Sutton says: "In a fair proportion of cases, (20 percent) epithelioma of the tongue is preceded by changes known as leucoplakia and ichthyosis, and they are frequently referred to as pre-cancerous conditions. In the case of the cheek, epithelioma is sometimes preceded by a patch of leucoplakia. The disease often starts close to the angle of the mouth and extends backward into the cheek; or it begins in the fold of mucous membrane between the gum and the cheeks, and occasionally it starts in the center of the cheek, often on a level with the meeting place of the crowns of the upper and lower molar teeth."

Senn, in speaking of carcinoma of the mouth, says: "Carcinoma of the mucous membrane of the cheek is sometimes preceded by a patch of leucoplakia. The influence of chronic irritation in producing carcinoma is well shown in carcinoma in this locality, as the tumor very often corresponds in its location with the crowns of upper and lower molar teeth."

Butlin states that out of eighty cases of the tongue sixteen were preceded by leucoma.

Park believes leucokeratosis may become the seat of an epithelioma and its surgical interest depends upon the frequency with which it is followed by this malignant growth.

Warren says he has seen but few cases of leucoma; one of these in a lady, on whose tongue it first appeared in youth and remained in the shape of several large, brilliant white patches until old age, when it disappeared. In another case, a man forty-three years of age, the tongue had been troublesome from childhood; the mucous membrane was sensitive and easily irritated, and it was prone to inflamatory conditions, during which small ulcers appeared. At the age of thirty-four typical leucoma appeared, situated, for the most part, on the right side of the tongue. Three years later the patches enlarged and a warty growth formed in the center. Three years later he (Warren) removed with the knife the largest patch, which was about the a size of a silver dollar. This operation was performed in June, 1891. In October, 1891, a small epithelial growth of an apparently malignant nature appeared on the opposite side of the tongue. This growth was removed and found to be a typical cancer. In December a similar growth was removed from the tip of the tongue. In April, 1892, both growths having reappeared, a large portion of the left side and the tip of the tongue were removed by a wedge-shaped incision. The disease never returned in the tongue, but six months afterwards a glandular enlargement was observed under the left jaw, and the patient died two months later. The growth was found to be a typical carcinoma.

Treatment: Leucoplakia of the oral mucous membrane is generally rebellious to treatment, and quite often shows a marked tendency to carcinomatous degeneration; therefore the measures employed are, *per force*, largely those of palliation and heroic operations.

These cases, however, which give a clear history of syphilitic infection may be benefited by a course of anti-syphilitic treatment; but it may be stated, as a fact, that up to the present time no drug has been discovered which, acting constitutionally, has any beneficial effect whatever upon the progress of the disease.

The preventive measures which may be instituted in the treatment of leucoplakia are the removal or discontinuance of all forms of chemical and mechanical irritation. Persons who suffer from an irritable and sensitive oral mucous membrane should avoid chemical irritants of whatever nature, particularly alcohol, in any of its forms, acids, pungent condiments, very hot drinks and tobacco.

In persons already afflicted with the disease such irritants stimulate the progress of the disease, and should therefore be strictly interdicted. It is much easier, however, to advise a patient as to what he should do and what he should not do than it is to get him to follow your advice.

In the early stage of the disease—before it has caused any inconvenience—it is very difficult to get a man who is in the habit of using spiritous liquors or tobacco to consent to give them up. He feels that you may be mistaken, or that you are magnifying the danger, and hence decides not to change his habit of living, at any rate for the present, or until he is convinced that your advice is correct. Perhaps he will consult some other professional gentleman who disagrees with your diagnosis and laughs at your fears. This reassures the patient, and he who goes on with his old habit of life for months, perhaps years, in some cases with impunity, in others with most disastrous effects to his comfort and his life.

The mechanical irritants which are most common in the mouth are usually associated with the teeth or with artificial dentures, such as carious cavities, jagged roots, fractured teeth, salivary calculus, rough or ill-fitting plates, or plates made of materials which are irritating to a sensitive mucous membrane. All such forms of irritation should be at once removed by filling the cavities, extracting the roots, giving appropriate treatment to the fractured teeth, removing the salivary calculus and carefully polishing the surfaces of the teeth, while the irritating artificial dentures should be replaced by others, free from these objections, or discarded altogether. Too much stress cannot be laid upon these points as a safeguard to the patient against the development of the malignant form of the disease.

Local Treatment: Nicholson, who believes leucoplakia to be a zona of the oral mucous membrane, considers local applications of only temporary service, while the constitutional treatment for zona is often entirely fruitless. He desires, however, a trial of the tincture ferri perchloride 25 to 30 minims (1.3 to 2 gramms) three times per diem, as in one of his cases it seemed to give relief from the burning pain and improved the condition of the lingual epithelium in a remarkable manner, when all else had failed.

Rosenberger recommends the local application of pure balsam of Peru painted upon the patches with a brush, and allowing it to remain in contact for from three to five minutes. The immediate effect is a slight burning sensation with an abundant salivation. These applications he advises to be made three times per diem. In thirteen cases so treated great relief was obtained. The patches, however, heal slowly, a year in some cases being required to produce a cure.

Leistikow advises the local application of the following paste:

| | | | |
|---|---|---|---|
| Tenae silicae | 1.5 | gramms | (24 grains.) |
| Resorcini | 3.0 | " | (48 " ) |
| Adipis | 0.5 | " | ( 8 " ) |

This he applies to the affected parts with a swab. From eight to fourteen days afterwards a contraction or shriveling is observed and a slightly inflamed condition of the mucous membrane which, by the application of balsam of Peru, is brought to a normal condition.

Rosenberg reports a case of leucoplakia which had lasted for over seven years, and had resisted all the usual methods of treatment in which the plaques disappeared in a few days after being painted with a 20 percent solution of the iodide of potassium.

In case two, presented by the essayist, the local application to the plaques and the denuded surface of the tongue of tincture of aconite and tincture of iodine, equal parts, every other day for two months, relieved all the symptoms except the abnormal dryness of the tongue, while the denuded part healed and was covered with healthy appearing papillae. In the other four cases treatment was declined because of the apparent trivial nature of the disease to the mind of the patients.

Palliative Treatment: Consists of the use of alkaline lotions or mouth washes. Butlin recommends for this purpose, in the milder cases, bicarbonate of potassium fifteen to twenty grains to one ounce of water, and in the syphilitic cases chromic acid one to two grains to the ounce of water, or a five to ten grain solution may be painted upon the plaques. Bicyanide of mercury is also recommended in solution of one to two grains to an ounce of water and painted upon the plaques.

In the severer cases he recommends solutions of the bicarbonite of soda or of boracic acid. He thinks melboracis (honey and borax) is better suited to some cases than alkaline solutions, but, as a general rule, the alkaline solutions give greater relief in cases of leucoplakia of long standing. A trial of these various remedies is necessary in order to find the one best suited to the individual case.

Surgical Treatment: In the severer forms of the disease radical operation is the only safe method to follow. The tendency of the disease to assume a malignant character should cause it to be treated as a malignant growth and thorough extirpation practiced at the earliest moment. Temporizing by the use of caustics is worse than useless, and most authors depricate their use for the reason that the irritation seems to increase the dangers from malignant degeneration.

Garretson was very emphatic in his denunciation of the use of caustics of every form in the treatment of this disease.

Butlin says: "One general rule holds good for all cases of leucoma, namely: not to use caustics. Whatever danger there may be of the development of carcinoma is certainly increased by the employment of nitrate of silver and other caustics."

In the fatal case of leucoplakia buccalis and gingevae reported by the essayist, the physician who had charge of the case treated it with nitrate of silver and later with chromic acid, with the result of stimulating the more rapid spread of the disease. On the other hand, we may contract the results obtained by a radical operation in an almost identical case, also referred to on a previous page, in which a permanent cure resulted, as proved by the fact that there has been no recurrence after a period of over eight years. The concensus of opinion obtained from a perusal of the most eminent authorities is, that thorough and complete extirpation of the diseased tissue, is the only reliable method of treatment, and this, to be most effective, must be practiced before malignant symptoms have developed.

Perrin, who reports a case of leucoplakia linguae and labialis with papillematous epithelial degeneration, secured a permanent recovery by the thorough extirpation of the plaques by surgical means. He urges early and complete extirpation as the only way by which to avoid a final transformation of the disease into true epithelial carcinoma.

Dubois-Havenith has exhibited a case of leucoplakia linguae upon the left border of the tongue, which was successfully treated by curetting and the galvano-cautery.

Butlin does not recommend the early excision of the plaques when the disease is located in the tongue unless it "is very obstinate and scarcely at all relieved by treatment"; but he has no doubt of the wisdom of such an operation in "indurations, warty growths and very obstinate ulcers, particularly when they present the slightest increase of induration about their bases. Such conditions must be considered as young cancers, and must be dealt with as if they were, in truth, cancers." Hulke urges early excision of all hard and warty patches (ichthyosis) upon the tongue before they attain a large size as the only means of permanent cure.

In closing this somewhat lengthy paper your essayist desires to thank you for your forbearance and kindness in listening so attentively, and trusts that the presentation of such a subject has not seemed to you out of place, but rather in its right place, and that the suggestions herein made will have so interested you that in the future you will carefully observe the mouths of your patients with the view of studying this disease and reporting your discoveries. The disease is, no doubt, on the increase, if your essayist may judge from his own experience, for up to one year ago he had seen but three cases in all his practice; since this period he has recorded five additional cases.

BIBLIOGRAPHY.

Zeigler.—Text-Book on Pathology, Anatomy and Pathogenisis.

Park.—Surgery by American Authors.

Warren.—Surgical Pathology and Therapeutics.

Sutton.—Tumors, Innocent and Malignant.

Senn.—Pathology and Surgical Treatment of Tumors.

Butlin.—Diseases of the Tongue.

Hyde.—Diseases of the Skin.

American Text-Book of Surgery.

Sarjous.—Annals, 1889 to 1896.

Rosenberger.—Munchner Medicinische Wochenschrift, October 30, 1889.

Garretson.—International Dental Journal, June, 1890.

Shield.—British Medical Journal, November 29, 1890.

Du Castel.—Annales de Dermatologie et de Syphilography, Paris, page 182, 1892.

Perrin.—Marseille-medical, November 15, 1892.

Dubois-Havenith.—Journal de Medicine, de Chirurgie et de Pharmacologie, Buogie, Bruxelles, January 23, 1894.

Nicholson.—British Medical Journal, March 6, 1894.

Le Dentu.—Practitioner, London, March 1895.

Rosenberg.—Deutsche Medicinische Wochenschrift, Leipsic, September 13, 1894.

Erb.—Munchener Medicinische Wochenschrift, Munich, October 18, 1892.

Hulke.—Translations of the Clinical Society of London, 1884.

